# Characterization of the complete chloroplast genome of *Begonia handelii*

**DOI:** 10.1080/23802359.2022.2119107

**Published:** 2022-12-12

**Authors:** Xiaozhen Zhu, Xinghua Hu, Shixun Huang, Qifeng Lu, Ting Liao

**Affiliations:** Guangxi Institute of Botany, Guangxi Zhuang Autonomous Region and Chinese Academy of Sciences, Guilin, Guangxi, China

**Keywords:** *Begonia handelii*, chloroplast genome, high-throughput sequencing, phylogenetic analysis

## Abstract

Begonia is the fifth largest genus of angiosperms in the world, and *Begonia handelii* is a member of the Begonia(Begoniaceae), and is one of the few species with floral fragrance in this genus. However, the chloroplast genome structure and phylogenetic relationship of this species is still unclear. In this study, the chloroplast genome of *B. handelii* was sequenced by Illumina HiSeq X platform, and the phylogenetic relationship of this species in Begonia was analyzed with related species. The whole chloroplast genome of *B. handelii* is 169,406 bp in size, which consist one large single-copy region (LSC) with 95,403 bp, one small single-copy region (SSC) with 20,089 bp, and two inverted repeat regions (IR) with 26,957 bp. The GC content of this chloroplast genome is 35.6%. Moreover, 140 genes were found in the chloroplast of *B. handelii*, including 90 protein-coding genes, 8 rRNA genes, 42 tRNA genes. Phylogenetic analysis showed that *B. handelii* is closed to *B. coptidifolia* and *B. pulchrifolia*. This study lays the foundation for further research on the chloroplast genome evolution of *B. handelii* chloroplasts.

*Begonia* is the fifth largest vascular plant genus in the world, and more than 2,000 species have been officially published worldwide (Moonlight et al. 2019). *Begonia* is one of the most diverse ornamental plants in the world (Tian et al. 2020, Li et al. 2021). *Begonia handelii* Irmsch (1921) is one of the few species with floral fragrance and dioecious in *Begonia* (Ding et al. 2021), which has beautiful flowers and leaves that has great potential in horticultural development. Its distribution is limited to Yunnan, Guangxi and Guangdong in China, as well as Vietnam. It grows in the shady and damp place in the dense forest by the roadside on the hillside, at an altitude of 150–850 m. The leaves of *B. handelii* are broadly oval in outline, with short awns when young. The complete chloroplast sequence of this species has not been reported. This study reports the chloroplast genome sequence of *B. handelii*, which contributes to provide more chloroplast genome genetic information in *Begonia*, and provide some information for the study of Begonia phylogeny and evolution.

The fresh leaves of *B. handelii* were collected at Guangxi Institute of Botany, Guangxi Zhuang Autonomous Region and Chinese Academy of Sciences (110°18′2″ E, 25°4′50″ N). *B. handelii* sampling was permitted by the National Natural Science Foundation of China (project number 32060025). A specimen was stored at the Herbarium of Guangxi Institute of Botany (http://www.gxib.cn/spIBK/, Z. C. Lu, email: zhaocenlu@163.com) under the voucher number IBK 00438290. The total genomic DNA was extracted by a DNeasy Plant Mini Kit (Qiagen, USA) and sequenced on the Illumina HiSeq X platform (Illumina, San Diego, CA, USA). Finally, there were approximately 3.86 Gb of clean data generated using paired-end sequencing method. The chloroplast genomes of *B. handelii* were assembled using NOVOPlasty 4.2.1 (Dierckxsens et al. 2017). Subsequently, the chloroplast genome were genetically annotated using the CPGAVAS software (Zuo et al. 2017). Finally, the assembled chloroplast genome and its detailed annotations were submitted to GenBank under the accession number OM287194.

The assembled chloroplast of *B. handelii* is 169,406 bp in length, with 35.57% GC content. The genome contains a large single-copy region (LSC) of 95,403 bp, a small single-copy region (SSC) of 20,089 bp, which separated by a pair of inverted repeat regions (IRA and IRB) of 26,957 bp. A total of 140 genes were identified, including 90 protein-coding genes, 8 rRNA genes and 42 tRNA genes. Among these genes, twenty genes contain one intron (*trnI-GAU, ndhB, rpl2, trnK-UUU, trnG-UCC, atpF, rpoC1, petB, petD, rpl16, rps16, ndhB, ndhA, trnA-UGC, trnG-UCC, trnL-UAA, trnV-UAC, trnK-UUU, trnI-GAU.* and *trnA-UGC*), while three genes (*rps12, ycf,.* and *clpP*) have two introns.

We construct a phylogenetic tree based on the complete chloroplast genome of *Begonia* downloaded from NCBI, using three species of Cucurbitaceae as outgroups. The phylogenetic tree was constructed using the maximum-likelihood method with 1000 bootstraps under the GTRGAMMAI substitution model (Coughenour et al. 2011). It was carried out using MEGA version 7 (Sudhir et al. 2016). Phylogenetic analysis revealed that *B. handelii* and *B. coptidifolia*, *B. pulchrifolia*, *B. versicolor*, *B. guangxiensis* gathered on a branch, and then *B. handelii* is closed to *B. coptidifolia* and *B. pulchrifolia* (Figure 1).

**Figure 1.**
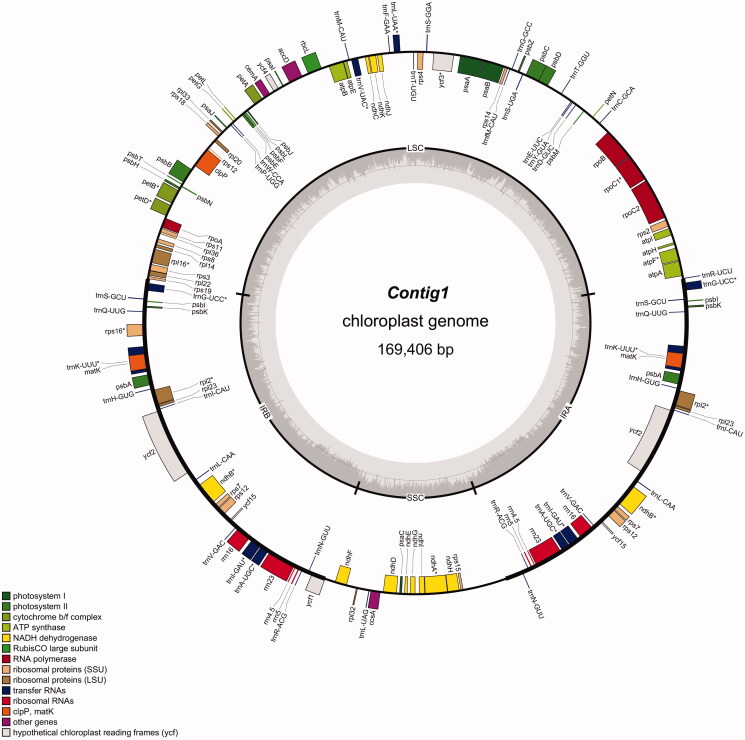
The phylogenetic relationship of *Begonia handelii* reconstructed with complete plastid genome sequences of *Begonia* downloaded from NCBI. Bootstrap support values >75% are indicated next to the branches.

**Figure 2.**
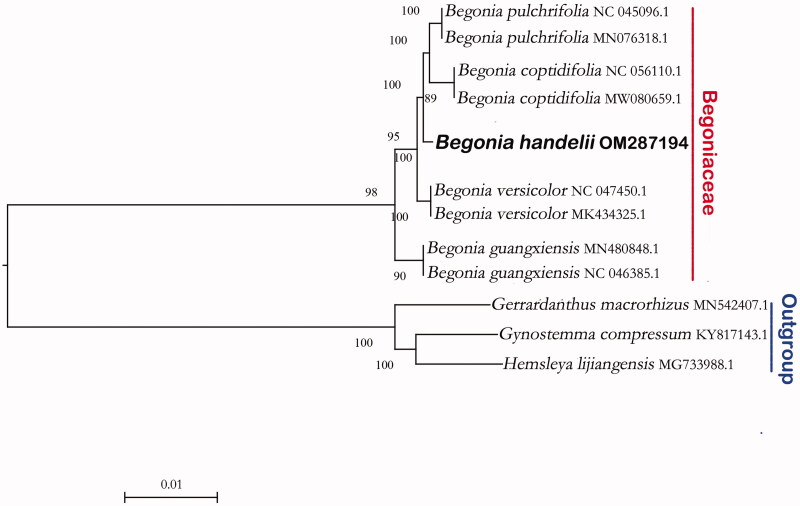
Chloroplast genome map of *Begonia handelii* (Wang et al. 2021).

## Data Availability

The complete chloroplast genome sequence of *B. handelii* has been deposited in GenBank with accession number OM287194, and is also accessible at https://www.ncbi.nlm.nih.gov/. The associated Bio-Project, SRA, and Bio-Sample numbers are PRJNA826728, SRR18788335, and SAMN27591385 are accessible at https://www.ncbi.nlm.nih.gov/. The data are ethically correct and do not violate the protection of human subjects or other valid ethical, privacy or security concerns. Chloroplast gene classification table of *Begonia handelii* (Zuo et al. 2017).
